# Plastid methylerythritol phosphate pathway participates in the hypersensitive response-related cell death in *Nicotiana benthamiana*

**DOI:** 10.3389/fpls.2022.1032682

**Published:** 2022-10-26

**Authors:** Sanghun Lee, Sung Hee Jo, Chi Eun Hong, Jiyoung Lee, Byeongjin Cha, Jeong Mee Park

**Affiliations:** ^1^ Plant Systems Engineering Research Center, Korea Research Institute of Bioscience & Biotechnology (KRIBB), Daejeon, South Korea; ^2^ Department of Plant Medicine, Chungbuk National University, Cheongju, South Korea; ^3^ Biological Resource Center, Korea Research Institute of Bioscience & Biotechnology (KRIBB), Jeongeup, South Korea

**Keywords:** bax-mediated cell death, chloroplast, hypersensitive response, LytB, methylerythritol phosphate pathway, reactive oxygen species

## Abstract

Programmed cell death (PCD), a characteristic feature of hypersensitive response (HR) in plants, is an important cellular process often associated with the defense response against pathogens. Here, the involvement of *LytB*, a gene encoding 4-hydroxy-3-methylbut-2-enyl diphosphate reductase that participates in the final step of the plastid methylerythritol phosphate (MEP) pathway, in plant HR cell death was studied. In *Nicotiana benthmiana* plants, silencing of the *NbLytB* gene using virus-induced gene silencing (VIGS) caused plant growth retardation and albino leaves with severely malformed chloroplasts. In *NbLytB*-silenced plants, HR-related cell death mediated by the expression of either the human proapoptotic protein gene *Bax* or an *R* gene with its cognate *Avr* effector gene was inhibited, whereas that induced by the nonhost pathogen *Pseudomonas syringae* pv. *syringae* 61 was enhanced. To dissect the isoprenoid pathway and avoid the pleiotropic effects of VIGS, chemical inhibitors that specifically inhibit isoprenoid biosynthesis in plants were employed. Treatment of *N. benthamiana* plants with fosmidomycin, a specific inhibitor of the plastid MEP pathway, effectively inhibited HR-related PCD, whereas treatment with mevinolin (a cytoplasmic mevalonate pathway inhibitor) and fluridone (a carotenoid biosynthesis inhibitor) did not. Together, these results suggest that the MEP pathway as well as reactive oxygen species (ROS) generation in the chloroplast play an important role in HR-related PCD, which is not displaced by the cytosolic isoprenoid biosynthesis pathway.

## 1 Introduction

Hypersensitive response (HR), characterized by programmed cell death (PCD) in plants, is often observed during the response to pathogen attack. Reactive oxygen species (ROS) generation, ion flux alterations, protein modifications, and gene activation have been implicated in the regulation of pathogen-induced PCD (Dangl et al., 1996; Greenberg and Yao, 2004; Van Breusegem and Dat, 2006; Mur et al., 2008; Huysmans et al., 2017). One of the most rapid plant defense responses to pathogen attack is ROS burst, which involves the production of superoxide anion ( ${\text{O}}_{2}^{•-}$ ), hydroxyl radical (•OH), and hydrogen peroxide (H_2_O_2_) at the site of attempted invasion (Byczkowski and Gessner, 1988; Apostol et al., 1989; Levine et al., 1994; Ausubel, 2005; Ye et al., 2021). Elevated ROS levels directly lead to pathogen death, thus strengthening the cell wall and acting as a signal to activate further defenses (Jabs, 1999; Torres et al., 2006). ROS are primarily generated by nicotinamide adenine dinucleotide phosphate (NADPH) oxidase in the plasma membrane (Doke et al., 1996). Other sources of ROS in plants include photosynthesis, photorespiration, and oxidative mitochondrial respiration (Foyer and Noctor, 2009). In plant cells, the chloroplast is a major source of ROS and therefore is an important player in cell death responses (Zapata et al., 2005). For example, in Tobacco mosaic virus (TMV)-infected leaves of tobacco (*Nicotiana tabacum*) plants, light induces H_2_O_2_ generation and cell death upon mitogen-activated protein kinase (MAPK) activation (Liu et al., 2007). Similarly, *Arabidopsis thaliana* plants grown under low light exhibit compromised local and systemic defense responses against infection by bacterial pathogens (Genoud et al., 2002). Light is required for the *HRT* (Hypersensitive Response to Turnip crinkle virus (TCV)) gene-mediated HR and resistance to TCV (Chandra-Shekara et al., 2006). Accelerated cell death 2 (ACD2), a chloroplast protein, modulates cell death and resistance to pathogen infection in *Arabidopsis* (Yao and Greenberg, 2006). Together, these findings suggest a link between chloroplast-derived signals and pathogen-induced PCD, although the nature of the link remains unknown.

Owing to extensive investigation, the signaling pathways of apoptosis are relatively well known in animals (Willis et al., 2003). The B cell lymphoma-2 (Bcl-2) family consists of both cell death-inducing proapoptotic proteins (e.g., Bax, Bak, Bid, Bik, Bcl-XS, Bok, and Hrk) and cell survival-promoting antiapoptotic proteins (e.g., Bcl-SL, Bcl-2, Bcl-W, Bcl-XL, Bfl-1, Mcl-1, Brag-1, and A1) (Hockenbery et al., 1993; Green and Reed, 1998; Gross et al., 1999). Proapoptotic proteins induce the loss of mitochondrial membrane potential, which results in the release of several apoptotic regulatory proteins, including cytochrome *c* and procaspases, as well as ROS generation. For example, translocation of Bax to the outer mitochondrial membrane is accompanied by the release of apoptogenic factors, such as cytochrome c, into the cytosol, leading to the activation of caspases, a family of cysteine proteases that control and mediate the apoptotic response (Liu et al., 1996; Jurgensmeier et al., 1998). Genomic data analysis suggests that the animal Bcl-2 gene family does not exist in plants, which implies that plants possess different PCD pathways than animals (Ameisen, 2002). However, overexpression of the animal apoptosis protein gene *Bax* in plants can trigger HR-like apoptosis (Kawai-Yamada et al., 2001). Moreover, in tobacco (*Nicotiana benthamiana*), Bax-induced PCD was accompanied by an increase in ROS production and *Pathogenesis-Related* (*PR*) gene expression, similar to that observed during TMV-induced HR (Lacomme and Santa Cruz, 1999; Kawai-Yamada et al., 2001). In addition, *Arabidopsis* possesses a functionally conserved homolog of the mammalian Bax Inhibitor-1 (BI-1) protein, which inhibits PCD induced by Bax in yeast and plant (Kawai et al., 1999; Kawai-Yamada et al., 2001). BI-1 is an evolutionarily conserved transmembrane protein present in the endoplasmic reticulum (ER) that, when overexpressed in plants, inhibits various PCDs induced by pathogens, heat stress, and phytotoxins, in addition to Bax (Watanabe and Lam, 2008). These results suggest that some parts of the animal and plant PCD pathways are evolutionarily conserved at the molecular level. In the past few years, BI-1 has been identified as a novel apoptosis regulatory factor belonging to the TMBIM (Transmembrane BAX Inhibitor-1 Motif-containing) protein family (Rojas-Rivera and Hetz, 2015). In mammals, proteins in this group have at least six highly conserved members, and in plants, several homologs of these proteins, in addition to BI-1, have been reported (Rojas-Rivera and Hetz, 2015). Although how BI-1 functions in cytoprotection is not yet clear, it is known to regulate calcium secretion from the ER, inositol-requiring enzyme 1 (IRE1) activity, and autophagy during ER stress in both plant and animals (Lebeaupin et al., 2020).

In the late 1990s, a new group of conserved eukaryotic apoptosis regulatory proteins, Bcl-2 associated athanogenes (BAGs), was discovered (Takayama et al., 1995; Takayama and Reed, 2001). BAGs have a common conserved region that interacts with the ATPase domain of heat shock protein 70 (Hsc70/Hsp70), called the BAG domain (BD), which is known to be involved in PCD and autophagy in various stressful environments (Takayama and Reed, 2001). Advanced bioinformatics analysis, based on protein structure rather than on primary sequence, has revealed that *Arabidopsis* possesses seven BAG homologous genes containing the BD (Doukhanina et al., 2006). BAG homologs are also present in other plant species and, similar to those in animals, are cytoprotective against a variety of agents that induce stress-induced PCDs, including pathogens (Williams et al., 2014). Plant BAGs, unlike animal BAGs, are localized to various subcellular organelles, and some BAGs have a calmodulin binding domain not found in animal BAGs. These results, together with the observed presence or absence in plants of other major animal apoptosis regulators, has led to speculation that the mechanisms of cell death regulation in plants are different from those in animals (Thanthrige et al., 2020).

Isoprenoids are found in all living organisms but are especially abundant and diverse in plants (Peñuelas and Munné-Bosch, 2005; Kirby and Keasling, 2009; Pulido et al., 2012). These compounds are produced from a basic five-carbon unit, isopentenyl diphosphate (IPP), and its isomer, dimethylallyl diphosphate (DMAPP). IPP and DMAPP are produced through one of two pathways: the cytosolic mevalonate (MVA) pathway and the chloroplast 1-deoxy-D-xylulose 5-phosphate (DXP)/2-C-methyl-D-erythritol 4-phosphate (MEP) pathway (Peñuelas and Munné-Bosch, 2005; Kirby and Keasling, 2009; Pulido et al., 2012). The MVA pathway has been extensively studied in various organisms, including yeast and animals; however, the MEP pathway was uncovered in plants only in recent decades. The MEP pathway, which comprises seven enzymatic steps, has been identified in eubacteria and photosynthetic organisms, including plants, algae, cyanobacteria, and diatoms (Banerjee and Sharkey, 2014). Genetic analyses revealed that the MEP pathway plays critical roles in multiple aspects of plant cell physiology (Phillips et al., 2008). However, many questions remain about the reciprocal network of isoprenoid biosynthetic precursors (IPP and DMAPP), which are synthesized independently in two intracellular compartments, the cytoplasm and the chloroplast, and the effect of these precursors on plant development. Nonetheless, no studies have yet been conducted to investigate the effect of the MEP pathway on pathogen-induced PCD in plants.

Previously, using the virus-induced gene silencing (VIGS) approach, we studied the HR involvement of pepper (*Capsicum annuum*) genes upregulated in response to biological stresses in *N. benthamiana* plants (Lee et al., 2013). Here, we report on another gene identified in the VIGS screen, which encodes 4-hydroxy-3-methylbut-2-enyl diphosphate reductase (LytB), an enzyme that catalyzes the last step of the plastid MEP pathway. Using a combination of genetic knockdown and chemical inhibition assays, we show that the MEP pathway is essential for Bax-mediated PCD as well as for HR-mediated R–Avr interaction. These findings provide important evidence showing that, in addition to ROS, the MEP pathway is involved in regulating plant HR-related PCD.

## 2 Materials and methods

### 2.1 Cloning of *NbLytB*

To isolate the *NbLytB* gene, a *N. benthamiana* DNA library was amplified by PCR using sequence-specific primers designed on the basis of an Expressed Sequence Tag (EST; GenBank accession no. AY497304). The PCR amplicon was purified using the Gel&PCR purification system (BioFACT, Daejeon, South Korea), and the purified product was cloned into the pGEM T-Easy vector (Promega, Madison, WI, USA). The cloned fragment was sequenced and then used to design primers for 5´-rapid amplification of cDNA ends (RACE) PCR. Then, total RNA was extracted from *N. benthamiana* leaves and amplified by 5´-RACE PCR, using the designed primers and Ambion’s FirstChoice^®^ RLM RACE Kit (Ambion, Austin, TX, USA), according to the manufacturer’s instructions.

### 2.2 VIGS assay

Wild-type (WT) tobacco (*N. benthamiana*) plants were grown in soil in an environmentally controlled growth room at 23°C under 16 h light/8 h dark photoperiod. VIGS was used to silence the target *N. benthamiana* genes as described previously (Liu et al., 2002; Moon et al., 2016). Briefly, recombinant *pTRV2* plasmids and the *pTRV1* vector containing RNA1, which is required for virus replication, were separately transformed into *Agrobacterium tumefaciens* strain GV2260. A 5 ml culture of *A. tumefaciens* containing each *pTRV2* construct and a 20 ml culture of *A. tumefaciens* harboring *pTRV1* were grown at 28°C for 16 h in Luria-Bertani (LB) medium (Thermo Fisher Scientific, Waltham, MA, USA) containing 100 µg/ml rifampicin (Sigma-Aldrich, St. Louis, MO, USA) and 50 µg/ml kanamycin (Sigma-Aldrich). To perform VIGS assays, *A. tumefaciens* cells were resuspended in infiltration buffer (10 mM MgCl_2_, 10 mM MES (pH 5.6), 200 µM acetosyringone (Sigma-Aldrich)) to a final optical density (OD_600_) of 0.5 (*pTRV2*) or 0.7 (*pTRV1*), and each *pTRV2* cell suspension was mixed with the *pTRV1* suspension at a 1:1 ratio. After a 4 h incubation at room temperature, the mixed *Agrobacterium* cultures were infiltrated into the abaxial surface of *N. benthamiana* leaves using a needleless syringe. A morphological phenotype of gene silencing was observed in systemic leaves at 10 days after infiltration. *Agrobacterium*-mediated transient expression experiments were performed on leaves with a relatively high probability of target gene silencing, based on their albino phenotype caused by silencing of the *NbPDS* or *NbLytB* gene.

### 2.3 Semiquantitative reverse transcriptase PCR

Total RNA was extracted from the agroinfiltrated *N. benthamiana* leaves using TRI Reagent (Molecular Research Center, inc., Cincinnati, OH, USA), according to the manufacturer’s instructions, and treated with DNase I (NEB, Ipswich, MA, USA). Then, cDNA was synthesized from 2 μg of total RNA using the Moloney murine leukemia virus (M-MLV) reverse transcriptase (Invitrogen, Carlsbad, CA, USA), according to the manufacturer’s instructions. Semiquantitative RT-PCR was performed on DNA Engine^®^ Peltier Thermal Cycler (Bio-Rad, Hercules, CA, USA) using AccuPower^®^ PCR PreMix (Bioneer, Daejeon, Korea) and gene-specific primers (**Supplementary Table 1**). The PCR program consisted of 30 cycles (for *phytoene desaturase* (*NbPDS*) and *NbLytB* genes) and 22 cycles (for the *NbActin* gene; internal reference) of the following three steps: 95°C for 30 s, 55°C for 30 s, and 72°C for 1 min. PCR products were separated on 1.0% agarose gels and visualized by ethidium bromide staining (Burton et al., 2000; Liu et al., 2002).

### 2.4 HR-PCD assays

*A. tumefaciens* cells carrying different expression constructs (*pBtex*:empty vector, *Bax*, *HRT*, *TCV CP*, *Rx*, *PVX CP*) were grown overnight in LB medium containing 100 µg/ml rifampicin and 50 µg/ml kanamycin. Subsequently, the *Agrobacterium* cells were precipitated, washed, and resuspended in infiltration buffer to obtain a final OD_600_ of 0.5. Then, the *Agrobacterium* cell suspensions were incubated at room temperature for 4 h before infiltration into *N. benthamiana* leaves. *Pseudomonas syringae* pv. *syringae* 61 (*Pss*61) and the *hrp^-^* mutant were grown overnight at 28°C in King’s medium (Duchefa Biochemie, Haarlem, Netherlands), and resuspended in 10 mM MgCl_2_ (10^4^ cfu/ml).

### 2.5 Pathogen inoculation

Viral inoculum was prepared from *N. benthamiana* leaves infected by GFP-tagged TMV (TMV : GFP). Two weeks after VIGS, *N. benthamiana* plants expressing the TMV resistance *N* gene were rubbed with the sap of leaves inoculated with TMV : GFP and containing carborundum (Sigma-Aldrich) (Collum and Culver, 2017). All inoculated plants were maintained in the growth room at 23°C under 16 h light/8 h dark photoperiod. TMV : GFP fluorescence in the inoculated plants was observed daily using a UVP BioImaging system (UVP LLC, Upland, CA, USA), and photographs were taken using a Nikon Coolpix E8800 digital camera (Nikon, Tokyo, Japan). Two independent biological replicates were performed, with each replicate containing at least three plants.

*Pseudomonas syringae* pv. *tabaci* (*Pst*) was grown overnight at 28°C in King’s B medium, resuspended in 10 mM MgCl_2_ (10^4^ cfu/ml), and infiltrated into the leaves of gene-silenced or inhibitor-treated plants using a 1 ml plastic needleless syringe. To measure the bacterial inoculum, the leaves of three independently silenced plants were harvested at 0, 1, 2, and 4 days post inoculation (dpi). Two 1.0 cm^2^ leaf disks were excised from each leaf, and leaf disks was ground in 1 ml of 10 mM MgCl_2_. Then, 100 µl of 10-fold serial dilutions of each leaf-disk extract was plated on King’s B-agar medium containing 50 µg/ml spectinomycin (Sigma-Aldrich), and the colonies formed were counted.

### 2.6 Trypan blue staining

The TB staining procedure was performed using modifications of a previously described method (Guo et al., 2019). Leaf samples were covered with lactophenol–Trypan blue (Sigma-Aldrich) mixture (10 ml of glycerol, 9.3 ml of unsaturated liquid phenol, 10 ml of lactic acid, and 10 mg of TB) and then subjected to vacuum infiltration for 5 min in a desiccator. The plate harboring leaf samples was placed in boiling water for 5 min and then incubated at room temperature for 12–16 h. After removing the staining solution, the leaves were covered with 2.5 g/ml chloral hydrate (TCI, Kasei Kogyo, Tokyo, Japan) for destaining. The destained leaves were equilibrated with 60% (w/v) glycerol, mounted on glass slides, and observed under a microscope (Zeiss, Munich, Germany).

### 2.7 Measurement of H_2_O_2_ levels

Production of H_2_O_2_ was detected, as described previously (Thordal-Christensen et al., 1997), using 3, 3’-diaminobenzidine (DAB) (Sigma-Aldrich), which forms a reddish-brown precipitate upon reaction with H_2_O_2_. Briefly, Bax-treated full leaves were detached at 0, 12, and 24 h postinfiltration; placed in 1 mg/ml DAB (pH 5.6); and subjected to vacuum infiltration for 10 min. Subsequently, the leaves were incubated at 23°C for 8 h under light. To remove chlorophyll and excess DAB stain, the leaves were boiled in alcohol: lactophenol (2:1) solution for 5 min. The destained leaves were rinsed three times with 50% ethanol and then stored in 50% ethanol. The DAB staining pattern and intensity were assessed visually (Samuel and Ellis, 2002).

### 2.8 Transmission electron microscopy analysis

Sections of leaves were obtained from 2-week-old plants subjected to VIGS and fixed for 2 h in 2.5% paraformaldehyde–glutaraldehyde mixture buffered with 0.1 M phosphate (pH 7.2). The fixed leaf sections were then postfixed for 1 h in 1% osmium tetroxide (OsO_4_) prepared in 0.1 M phosphate buffer (pH 7.2), and dehydrated in graded ethanol and propylene oxide. The dehydrated samples were embedded in Epon-812, and ultrathin sections were prepared using the UltraCut E ultramicrotome (Leica, Vienna, Austria). The prepared sections were stained with uranyl acetate and lead citrate (UA/Pb), and examined on a CM 20 electron microscope (Philips, Amsterdam, Netherlands).

### 2.9 RNA blot analysis

To perform RNA blot analysis, 10 µg of total RNA was separated electrophoretically on a 1% formaldehyde-containing agarose gel and then transferred onto a Hybond-N+ nylon membrane (GE Healthcare, Buckinghamshire, UK) by overnight capillary transfer with 20× SSC (3 M NaCl, 0.3 M sodium citrate (pH 7.0)). To determine TMV : GFP transcript levels, the RNA blot was hybridized with a 412 bp cDNA fragment of the *movement protein (MP)* gene of TMV, which was amplified using the *TMV MP*_F and *TMV MP*_R primers (**Supplementary Table 1**) and subsequently labeled with [^32^P]-dCTP using the Rediprime II DNA Labeling System (GE Healthcare) hybridization of the radiolabeled probe was performed as described previously (Church and Gilbert, 1984; Choi et al., 2002). Then, the membrane was subjected to two 5 min washes each with prewarmed 2× SSC and 0.1% SDS. The hybridization signal was visualized on a BAS-1800II PhosphorImager (Fuji Photo Film, Tokyo, Japan).

### 2.10 Chemical inhibitor treatments

Three inhibitors were used to perform the chemical inhibition assays: mevinolin (MEV; AG Scientific, San Diego, CA, USA), an inactive lactone, which inhibits 3-hydroxy-3-methylglutaryl coenzyme A reductase and thus blocks the cytosolic MVA pathway; fluridone (FLU; Duchefa), which inhibits PDS activity, thus blocking the carotenoid biosynthesis pathway; and fosmidomycin (FOS; 3-(N-hydroxyamino) propyl phosphate) (Invitrogen), which specifically inhibits (DXR) in the chloroplast. Ten millimolar stock solutions of MEV and FLU were prepared in ethanol and stored at 4°C, while that of FOS was prepared in water and stored at -20°C. To perform the chemical inhibitor treatments, working solutions of each inhibitor (100 µM MEV, 100 µM FLU, 1 mM FOS) were prepared from the corresponding stock solutions and infiltrated into entire abaxial surface of the fully expanded leaves of 4-week-old *N. benthamiana* plants using a 1 ml needleless syringe. Sixteen hours after the inhibitor/mock treatment, the inhibitor-treated leaves were infiltrated with elicitors (*Bax*, *HRT/TCV CP*, *Rx/PVX CP*) and nonhost pathogen (*Pss*61) for PCD and gene expression analyses.

### 2.11 Measurement of ion leakage

Four leaf disks (1 cm in diameter) were floated on 6 ml of distilled water for 12 h at room temperature, with shaking. After the 12 h incubation, the conductivity of the water was measured (value A) with a NeoMet conductivity meter (EC-470L; iSTEK, Seoul, Korea). Finally, the water containing leaf discs were autoclaved at 121°C for 5 min, cooled to room temperature, and then the conductivity of the water was measured (value B). Ion leakage (%) in each sample was calculated as follows: (value A/value B) × 100. Means and standard deviations of three independent biological replicates were calculated (Mittler et al., 1999).

### 2.12 Immunoblot analysis

To extract total proteins, leaf samples were ground in liquid nitrogen and resuspended in urea lysis buffer (8 M urea, 100 mM NaH_2_PO_4_, 10 mM Tris-HCl (pH 8.0)). The samples were then centrifuged at 13,000 rpm for 15 min at 4°C, and the supernatants were mixed with 5× SDS sample buffer. The extracted proteins were separated on 12% SDS-polyacrylamide gels and transferred to polyvinylidene difluoride (PVDF) membranes (Roche, Basel, Switzerland). To determine Bax abundance, the membranes were incubated first with anti-Bax antibody (Santa Cruz Biotechnology, Dallas TX, USA) and then with horseradish peroxidase-conjugated anti-rabbit IgG antibody (Santa Cruz Biotechnology). Finally, the Bax protein was detected using the Enhanced Chemiluminescence (ECL) Kit (Thermo Fisher Scientific).

### 2.13 Chlorophyll fluorescence measurements

Two weeks after VIGS, fully expanded leaves were detached from three *N. benthamiana* plants silenced for the indicated genes. The detached leaves were dark-adapted for 30 min, and then the maximum photochemical efficiency of photosystem II (PSII; Fv/Fm), the level of photoprotective quenching of fluorescence (1–qP, where qP is the level of photochemical quenching of PSII, also referred to as Fq′/Fv′), recovery of nonphotochemical quenching (NPQ), and light response curves of the quantum yield of PSII (*Φ*_PSII_) were measured as described previously (Choi et al., 2002). The experiments were performed at room temperature.

## 3 Results

### 3.1 Silencing of *NbLytB* suppresses Bax-mediated PCD in *Nicotiana benthamiana*

In previous study, silencing of the pepper *LytB* gene resulted in an albino phenotype, similar to the phenotype induced by silencing of the phytoene desaturase (*PDS*) gene (Liu et al., 2002), which is widely used as positive control in VIGS experiments; however, the silencing of these two genes had opposite effects on Bax-induced PCD (data not shown). This prompted us to further investigate why these two genotypes exhibiting similar defects in chloroplast development respond differently to Bax-induced cell death. First, the *LytB* gene homolog was isolated from the *N. benthamiana* cDNA library, and the cDNA fragment (737–1,147 nt) was cloned into the *pTRV2* vector (**Supplementary Figures 1** and **2**). The resultant construct as well as *pTRV2:GFP* (negative control) and *pTRV2:NbPDS* (positive control) (Moon et al., 2016) were used to perform VIGS experiments. Consistent with the results of previous VIGS studies using MEP pathway genes (Page et al., 2004), the *NbLytB*-silenced *N. benthamiana* plants exhibited growth arrest and their newly emerged leaves showed an albino phenotype and wavy edges. The albino leaf phenotype of *NbLytB*-silenced plants was very similar to that of *NbPDS*-silenced plants (**Supplementary Figure 3A).** Furthermore, semiquantitative RT-PCR showed that the endogenous *NbLytB* and *NbPDS* mRNA levels were greatly reduced in silenced plants (**Supplementary Figure 3B**), thus confirming the silencing of target genes.

**Figure 1:**
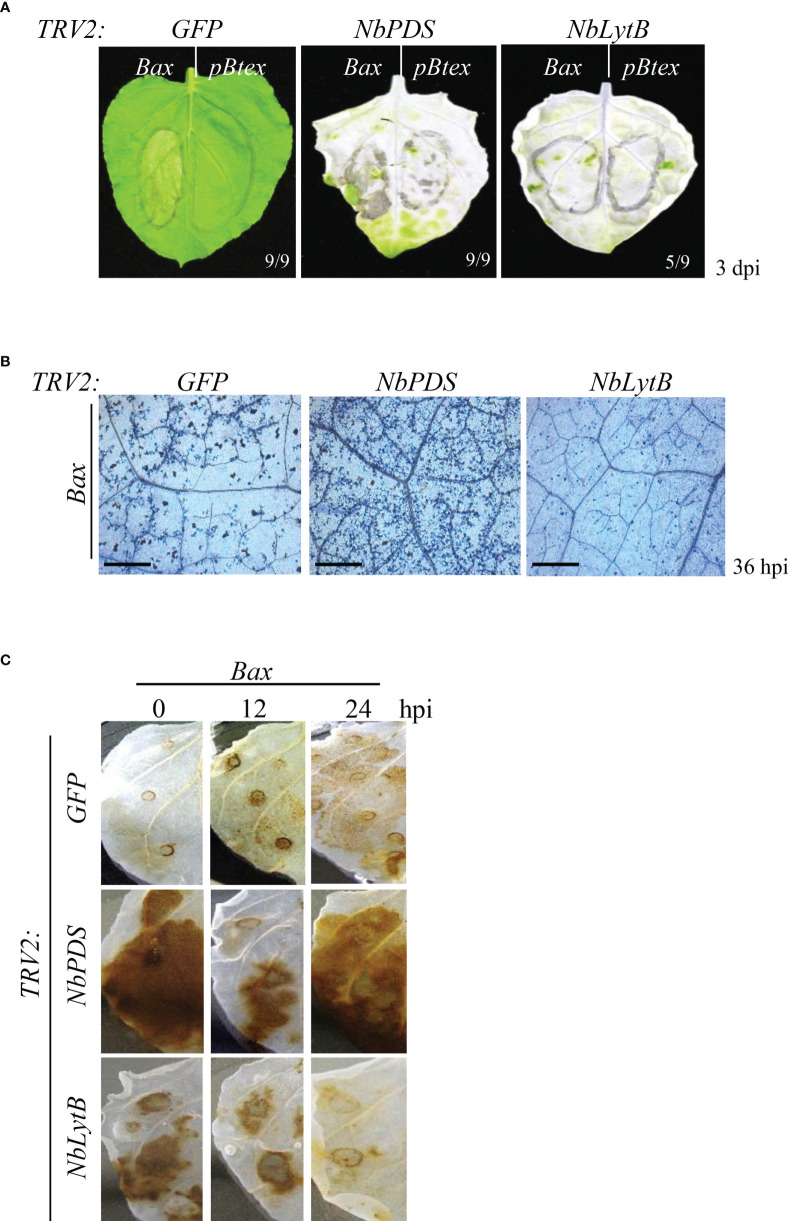
*NbLytB* is required for Bax-mediated PCD in *N. benthamiana* **(A)** Bax-mediated PCD in *TRV2*:*GFP* (control) plants, and *NbPDS*- and *NbLytB*-silenced plants. Areas infiltrated with *Agrobacterium* containing the empty *pBtex* vector or the *Bax* expression vector are outlined with a circle on each leaf. Pictures were taken 3 dpi. **(B)** Visualization of cell death in the leaves of *GFP*-, *NbPDS*-, and *NbLytB*-silenced plants by TB staining at 36 h after Bax infiltration. Scale bar = 20 µm **(C)** Impact of *NbLytB* on H_2_O_2_ accumulation by Bax-mediated PCD. H_2_O_2_ accumulation in *GFP*-, *NbPDS-*, and *NbLytB*-silenced leaves was visualized by DAB staining at the indicated time points after infiltration with *Agrobacterium* expressing *Bax*.

To determine the role of *NbLytB* in HR cell death, the human *Bax* gene was transiently overexpressed in *NbLytB*-silenced *N. benthamiana* plants through *Agrobacterium*-mediated transformation (Lee et al., 2013). In *GFP-* and *NbPDS*-silenced plants generated using the corresponding VIGS vectors, yellowing or necrosis was observed in all leaves at the site of *Bax* infiltration at 3 days post inoculation (dpi), whereas in the mock treatment site expressing the empty vector (*pBtex*), symptoms were mild or completely absent (**Figure 1A**). On the other hand, little or weak cell death was observed in the leaves of *NbLytB*-silenced plants (**Figure 1A**). In addition, cell death in silenced leaves was detected by staining each leaf with Trypan blue (TB) at 36 h after *Bax* infiltration (**Figure 1B**). Trypan blue enters cells with damaged membranes and binds to intracellular proteins, turning the cells blue (Koch and Slusarenko, 1990). Therefore, live (unstained) cells can be distinguished from dead (blue) cells under a microscope. Compared with the *GFP*-silenced (control) leaves, the number of dead (blue) cells was dramatically lower in *NbLytB*-silenced leaves and higher in *NbPDS*-silenced leaves (**Figure 1B**); this is consistent with the results shown in **Figure 1A**. Since the leaves of both *NbPDS-* and *NbLytB*-silenced plants showed the albino phenotype, we performed DAB staining to compare the ROS levels between the two genotypes (**Figure 1C**). ROS functions as a secondary messenger that mediates PCD in plants, and its accumulation is a hallmark of HR (Lamb and Dixon, 1997; Coll et al., 2011). The production of H_2_O_2_ in plant tissues can be visualized by staining with DAB, which is oxidized by H_2_O_2_, resulting in the formation of a brown precipitate (Thordal-Christensen, 1997). In *GFP*-silenced plants, the intensity of brown staining in the leaf area infiltrated with *Bax* was the strongest at 24 hours post inoculation (hpi) compared with the other time points (**Figure 1C**). On the other hand, the leaves of *NbLytB*-silenced plants showed the strongest brown staining immediately after *Bax* infiltration, and the level of H_2_O_2_ in these leaves was slightly lower than that in *GFP*-silenced leaves at 24 hpi. The *NbPDS*-silenced plants showed increased production of H_2_O_2_ at 0 and 24 hpi, and the level of H_2_O_2_ accumulation in *NbPDS*-silenced plants was considerably higher than that observed in *NbLytB*- and *GFP*-silenced plants. The strong brown color observed in *NbPDS-* and *NbLytB*-silenced plants immediately after agroinfiltration (0 hpi) was presumably caused by the production of H_2_O_2_ in response to wounding stress induced by agroinfiltration (Suzuki and Mittler, 2012) rather than by *Bax* expression. In addition, we suspect that the stronger DAB staining of both VIGSed plants relative to that of *GFP*-silenced control plants was because they contained malfunctioning chloroplasts that weakened their ROS scavenging function. Overall, the increase or decrease in cell death observed in *NbPDS*- and *NbLytB*-silenced plants, respectively, appears to be related to the generation of excess H_2_O_2_.

To determine whether *NbLytB* is also involved in plant pathogen-related PCD, cell death responses induced by nonhost pathogen infection or interaction between the *R* gene and its cognate effector were monitored in the silenced plants (**Supplementary Figure 3C**). In this experiment, the bean bacterial pathogen *Pss*61, which carries the *hrp* gene cluster and induces HR in *N. benthamiana* leaves (Alfano et al., 1997), was used as the nonhost pathogen, while its *hrp*-deficient mutant (*Pss*61*hrp* ^-^) was used as a non-HR induction control. Additionally, the *R* genes HRT and Rx (Resistance to Potato virus X (PVX)) were used in combination with their cognate effectors TCV coat protein (T-CP) and PVX CP (P-CP), respectively (Cooley et al., 2000; Bendahmane et al., 2002), while a CP mutant that did not induce *R* gene-mediated HR was used as a non-HR induction control (Moon et al., 2016). *GFP*-silenced plants (control) showed apparent cell death at 36 hpi in all PCD induction treatments (**Supplementary Figure 3C**). *NbLytB-*silenced plants suppressed Rx- and HRT-mediated HR, similar to the inhibitory response of Bax-induced cell death. On the other hand, in response to *Pss*61, the *NbLytB*- and *NbPDS*-silenced plants displayed a similar cell death phenotype to that of *GFP*-silenced control plants (**Supplementary Figure 3C**). These results indicate that the inhibition of PCD caused by *NbLytB* silencing is not common to all PCDs, and suggest that the role of chloroplasts in PCD may differ depending on the PCD elicitor.

#### 3.1.1 NbLytB is involved in both basal defense and *R* gene-mediated defense responses

**Figure 2:**
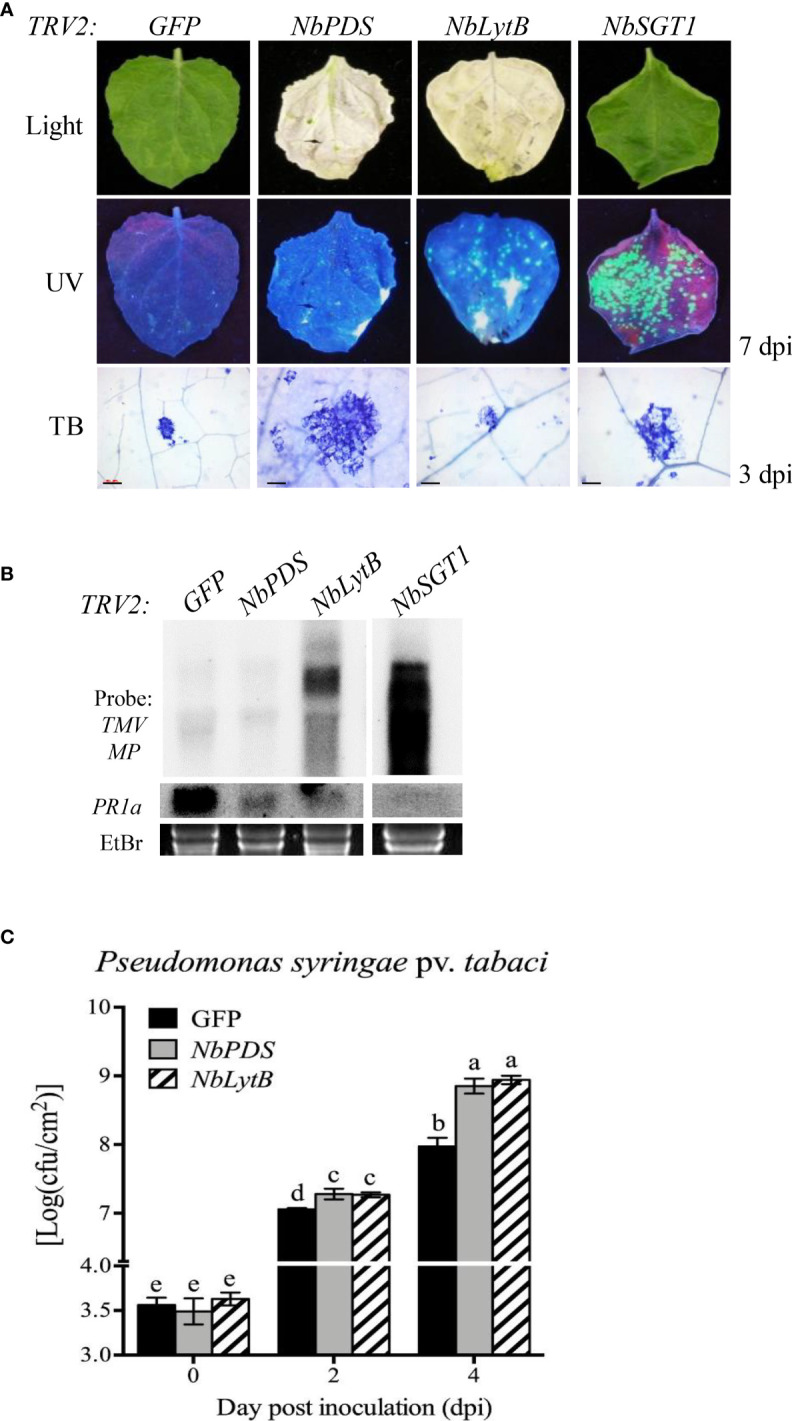
*NbLytB* silencing suppresses *N* gene-mediated resistance to TMV and increases the susceptibility of *N. benthamiana* plants to the bacterial pathogen *Pst* **(A)** Effect of NbLytB on the TMV *N* gene-mediated HR-PCD and on the resistance to TMV : GFP infection. Images show HR-PCD (top panel) and TMV : GFP fluorescence (middle panel) at 7 dpi. The leaves of *GFP*-, *NbPDS*-, *NbLytB*-, and *NbSGT1*-silenced transgenic *N. benthamiana* plants expressing the *N* gene were inoculated with TMV : GFP at 2 weeks after VIGS. HR-PCD was observed by TB staining at 3 dpi (bottom panel). Scale bars = 100 µm. *NbSGT1*-silenced plants were used as a positive control for TMV : GFP infection. WL, white light; UV, ultraviolet light. **(B)** RNA gel blot showing the accumulation of the MP of TMV : GFP and the expression of *PR1* in inoculated leaves collected at 6 dpi. The *rRNA* bands stained with ethidium bromide served as a loading control. Similar results were obtained in two independent experiments. **(C)** Growth of *Pst* in *GFP*-, *NbPDS*-, and *NbLytB*-silenced *N. benthamiana* plants. The leaves of gene-silenced plants were inoculated with *Pst*, and bacterial growth was evaluated at 0, 2, and 4 dpi. Data represent mean ± standard deviation (SD) of at least three independent biological replicates, with each replicate containing six leaf disks. Different letters indicate significant differences (*P* < 0.05; Student’s *t*-test). Three independent experiments were performed with similar results.

Next, we asked whether HR inhibition caused by the silencing of *NbLytB* was associated with the disease resistance response of plants. First, to observe the *R* gene-mediated disease resistance response, *N. benthamiana* plants carrying the TMV resistance gene *N* were subjected to VIGS to silence *NbPDS*, *NbLytB*, and *NbSGT1*, and then inoculated with GFP-tagged TMV (TMV : GFP) (Erickson et al., 1999; Liu et al., 2002). Silencing of *NbSGT1* was performed as a positive control to loss of TMV resistance by the *N* gene (Peart et al., 2002). At 7 days after TMV : GFP inoculation, fluorescent green spots were clearly observed in the inoculated leaves of *NbPDS*-, *NbLytB*-, and *NbSGT1*-silenced plants, and the size and number of these spots increased in the order of *NbSGT1*-, *NbLytB*-, and *NbPDS*-silenced plants. However, no GFP foci were observed in the inoculated leaves of *GFP*-silenced control plants (**Figure 2A**). TMV : GFP fluorescence was not observed in the upper leaves of all gene-silenced plants, except *NbSGT1*-silenced plants, at 14 dpi (data not shown). The *N* gene-mediated HR was observed in the inoculated leaves at 3 dpi *via* TB staining. Compared with the *GFP*-silenced control leaves, the size of TB-stained cells was increased in *NbPDS*- and *NbSGT1*-silenced leaves but slightly reduced in *NbLytB*-silenced leaves (**Figure 2A**). In addition, RNA gel blot analysis revealed virus accumulation in *NbLytB*- and *NbSGT1*-silenced plants (**Figure 2B**). These results indicate that NbLytB is potentially involved in HR and resistance responses mediated by the *N* gene.

To further evaluate the role of NbLytB in plant basal defense responses, the silenced leaves were inoculated with *Pseudomonas syringae* pv. tabaci (*Pst*), a disease-causing bacterial pathogen of *N. benthamiana* (Ramegowda et al., 2013). The *NbPDS*- and *NbLytB*-silenced plants showed 7–8-fold higher bacterial population than the *GFP*-silenced control plants (**Figure 2C**). However, no difference was observed in the degree of disease susceptibility between *NbPDS*- and *NbLytB*-silenced plants, suggesting that reduction in the basal resistance of these plants might be related to the lack of chloroplast function.

#### 3.1.2 Structural defects in chloroplasts and reduced photosynthetic efficiency in *NbLytb*-silenced plants

**Figure 3:**
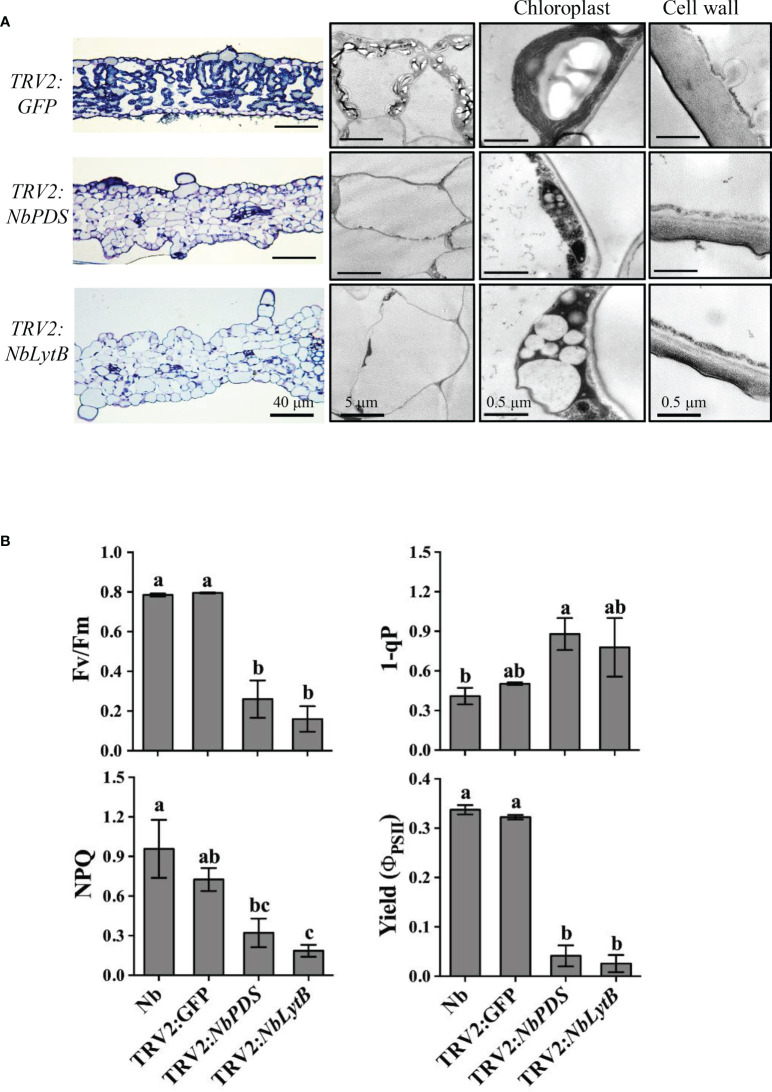
Downregulation of *NbLytB* disrupts chloroplast morphology and reduces photosynthetic efficiency. **(A)** Transmission electron microscope (TEM) images showing the ultrastructure of chloroplasts in the leaves of TRV2:*GFP* plants (control), and *NbPDS*- and *NbLytB*-silenced plants. Enlarged images show details of the chloroplasts and the cell wall. **(B)** Silencing of *NbLytB* reduces photosynthetic efficiency. The photosystem II (PSII) photochemistry of the detached leaves of unsilenced *N. benthamiana* (*Nb*) and *GFP-*, *NbPDS-*, and *NbLytB-*silenced *N. benthamiana* plants was examined by measuring the photochemical efficiency of PSII (Fv/Fm), reduced state of PSII (1-qP), nonphotochemical quenching of absorbed light (NPQ), and effective quantum efficiency of PSII (Yield). Data represent mean ± standard deviation (SD) of three replicates. Three independent experiments were performed with similar results.

To study whether the differences in HR between *NbPDS*- and *NbLytB*-silenced plants were caused by the differences in their chloroplast morphology, we examined the morphology of intracellular organelles in *NbPDS*-, *NbLytB*-, and *GFP*-silenced leaves by TEM (**Figure 3A**). The chloroplasts of *GFP*-silenced control plants showed a well-developed thylakoid membrane with large starch granules. However, in the albino sectors of *NbPDS*- and *NbLytB*-silenced leaves, chloroplasts were small in size, irregular in shape, and contained poorly developed thylakoid membranes, consistent with a previous report (Page et al., 2004). The chloroplasts of *NbLytB*-silenced leaves contained more vesicles than stroma and thylakoids (**Figure 3A**). In addition, the maximum efficiency of PSII photochemistry, i.e., the ratio of variable to maximal fluorescence (*Fv/Fm*), was drastically decreased in *NbPDS*- and *NbLytB*-silenced plants compared with the *GFP*-silenced control plants (**Figure 3B**). Taken together, these results indicate that HR inhibition by *NbLytB* silencing is not appreciably associated with chloroplast differentiation or a reduction in photosynthetic function.

#### 3.1.3 Suppression of HR by fosmidomycin

**Figure 4:**
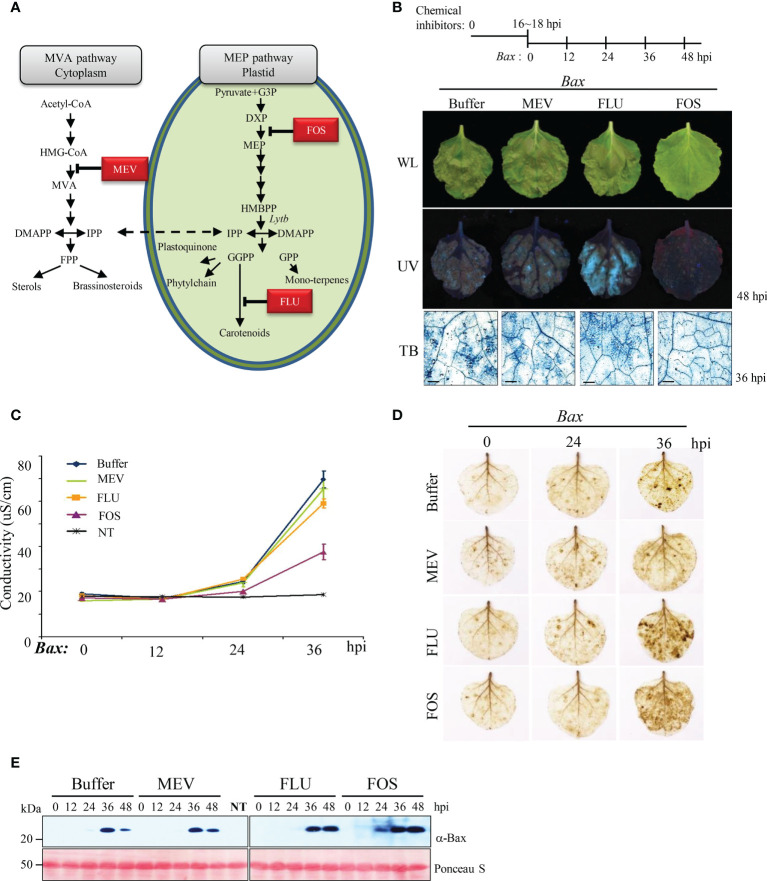
MEP pathway is involved in Bax-mediated PCD **(A)** Isoprenoid biosynthesis in plant cell. The first intermediate specific to each pathway is boxed. HMG-CoA, 3-Hydroxy -3-methylglutaryl CoA; MVA, mevalonic acid; IPP, isopentenyl diphosphate; DMAPP, dimethylallyl diphosphate; FPP, farnesyl diphosphate; GA-3-P, glyceraldehyde 3-phosphate; DOXP, 1-deoxy-D-xylulose-5-phosphate; MEP, 2-C-methyl-D- erythritol 4-phosphate; HMBPP, 1-hydroxy-2-methyl-2-**(E)**-butenyl 4-diphosphate; GGPP, geranylgeranyl diphosphate; GPP, geranyl diphosphate. The steps inhibited by MEV, FOS, and FLU are indicated. **(B)** Effect of selected inhibitors on Bax-mediated PCD. A schematic diagram of the experimental procedure used to study Bax-mediated PCD in plants pretreated with inhibitors of the MEP pathway is shown on the top. Pictures were taken under white light (WL; top panel) and ultraviolet light (UV; middle panel) at 48 hpi. Bax-mediated PCD was observed by TB staining at 36 hpi (bottom panel). Scale bar = 20 µm. **(C)** Ion leakage assay of leaves treated with chemical inhibitors and then infiltrated with Bax. Data represent means ± standard error (SE) of six leaf disks per genotype. NT indicates buffer-treated *N. benthamiana* without Bax infiltration. **(D)** DAB staining of inhibitor-treated *N. benthamiana* leaves at the indicated time points after infiltration with *Agrobacterium* expressing *Bax*. **(E)** Western blotting analysis of Bax expression in buffer- (control) and inhibitor-treated *N. benthamiana* leaves collected at 0, 12, 24, 36, and 48 hpi using anti-Bax antibody. Positive staining of inhibitor-treated leaves with anti-Bax antibody confirmed agroinfiltration with Bax. Rubisco (loading control) was detected by Ponceau S.

LytB catalyzes the last step of the MEP pathway in chloroplasts. To investigate the role of the MEP pathway in HR and rule out the pleiotropic effects of VIGS, we used chemical inhibitors that specifically inhibit the isoprenoid biosynthesis pathway in plants. Three chemical inhibitors were selected (FOS, FLU, MEV) (**Figure 4A**). FOS specifically inhibits DXR, an enzyme required to convert DXP to MEP in the chloroplast (Zeidler et al., 1998), which induces an overall inhibitory effect on chloroplast isoprenoid synthesis, as observed in *NbLytB*-silenced plants. MEV inhibits 3-hydroxy-3-methylglutaryl coenzyme A reductase, which blocks the cytosolic MVA isoprenoid biosynthesis pathway (Hemmerlin et al., 2003). Finally, FLU inhibits PDS activity and blocks the carotenoid biosynthetic pathway, inducing a *PDS*-silencing-like phenotype (Chae et al., 2004).

The treatment concentrations of FOS (1 mM) and FLU (100 µM) were adjusted to avoid plant cell damage and ensure that the yellowing of upper leaves caused by these treatments was similar (**Supplementary Figure 4A**). First, we monitored Bax-mediated HR cell death induced by each chemical inhibitor in *N. benthamiana* leaves. HR was clearly inhibited in FOS-treated leaves compared with the mock-treated (control) and other inhibitor-treated leaves (**Figure 4B**; **Supplementary Figure 4B**). These results were also confirmed through TB staining and quantitative analysis of ion leakage (**Figure 4B, C**). On the other hand, H_2_O_2_ production was not reduced in FOS-treated leaves, unlike VIGS, and the level of H_2_O_2_ in FOS-treated leaves was similar to that in FLU-treated leaves (**Figure 4D**). The accumulation of Bax protein in the chemical inhibitor-treated leaves was assessed by western blot analysis, which confirmed that Bax protein levels were not altered by inhibitor treatments (**Figure 4E**).

**Figure 5:**
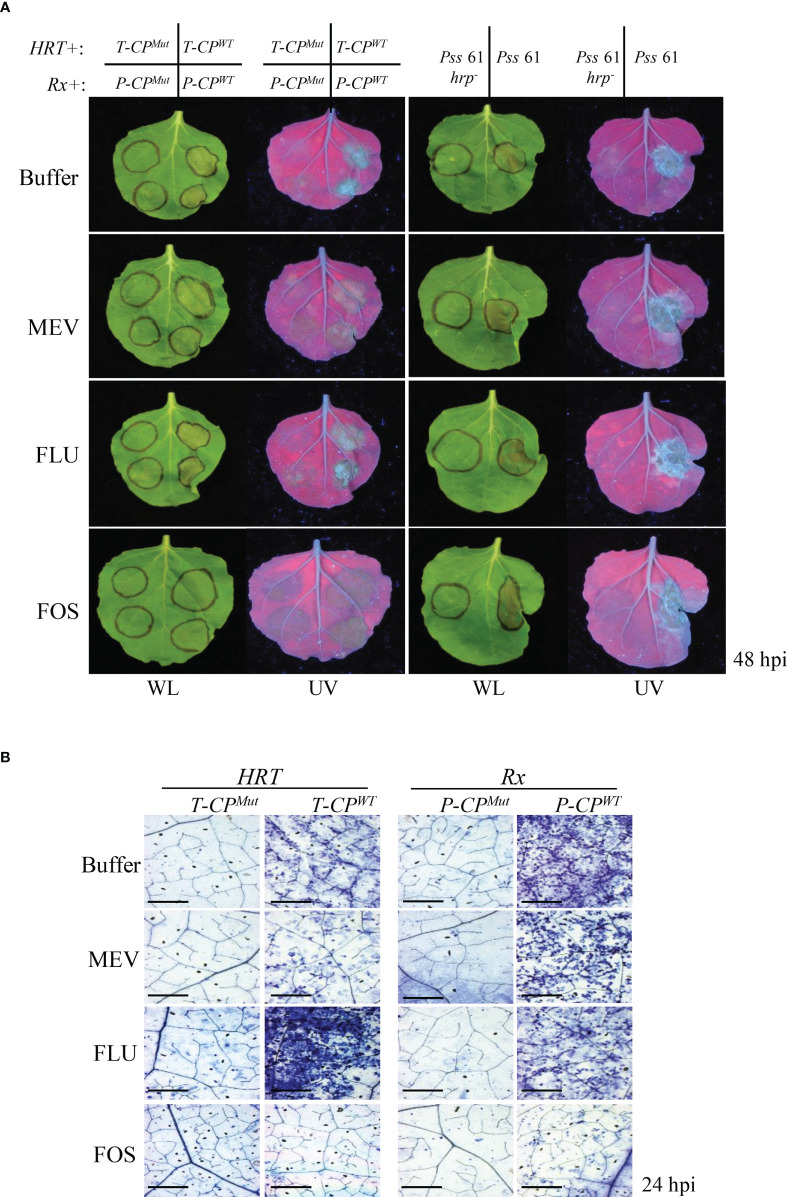
Inhibition of the MEP pathway suppresses *R*/Avr coexpression-mediated HR- related PCD **(A and B)** *R* gene-mediated and nonhost-induced plant PCD assays. **(A)** Left panel: *Agrobacterium* strains carrying the *R* gene were co-infiltrated with those carrying the corresponding *Avr* gene into *N. benthamiana* leaves 16 h after the buffer (control) or inhibitor treatment. T-CP^WT^,; T-CP^Mut^, mutant CP of TCV (negative control); P-CP^WT^; P-CP^Mut^, mutant CP of PVX (negative control). Right panel: bean (nonhost) bacterial pathogen *Pss*61 and its *hrp*-deficient mutant (*Pss*61*hrp^-^*; negative control) were inoculated into *N. benthamiana* leaves 16 h after the buffer (control) or inhibitor treatment. The PCD responses at the infiltration sites were evaluated at 48 hpi, and pictures were taken. WL, white light; UV, ultraviolet light. **(B)** Evaluation of cell death in the leaves of inhibitor-treated plants at 24 h after HRT/T-CP^WT or Mut^ or Rx/P-CP^WT or Mut^ infiltration by TB staining. The leaves of buffer-, MEV-, and FLU-treated plants showed numerous dead cells, whereas those of FOS-treated plants showed few dead cells. Scale bar = 20 µm.

Next, we examined whether the MEP pathway also modulates *R* gene-mediated HR-PCD and/or nonhost pathogen-induced PCD. Coexpression of HRT/T-CP^WT^ and Rx/P-CP^WT^ induced HR-PCD in the buffer-, MEV-, and FLU-treated plants but not in FOS-treated plants (**Figure 5A**). PCD induced by *Pss*61 was similar to the buffer-treated control in all inhibitor-treated leaves (**Figure 5A**). The *R* gene-induced PCD was further supported by the microscopic observation of the cell death response in TB-stained leaves (**Figure 5B**).

To examine how the chemical inhibitor treatment affects the basal plant defense response, the inhibitor-treated plants were inoculated with *Pst* and monitored for bacterial growth **(Supplementary Figure 4C**). Plants treated with FOS and FLU showed increased susceptibility to *Pst* compared to the mock- or MEV-treated plants, suggesting that chloroplast is essential for an effective basal resistance response to pathogens.

## 4 Discussion

In this dissection of the novel mechanisms of HR-related PCD in plants, the plant *LytB* gene was identified as being involved in Bax- and R–Avr interaction-induced HR cell death. Silencing of *NbLytB* in *N. benthamiana* plants efficiently suppressed Bax-mediated PCD and HR as well as defense responses against pathogens such as *Pst* and TMV : GFP. Consistent with the results of silencing *NbLytB*, the FOS-mediated inhibition of IPP biosynthesis in plastids interfered with Bax- and R–Avr interaction-induced PCD and defense responses following pathogen infection. These results reveal that the IPP metabolite in plastids plays critical roles in HR PCD associated with plant immunity.

VIGS utilizes the short interfering RNA (siRNA)-induced antiviral defense mechanism of plants and is considered a very suitable technique for studying the functional genomics of polyploid plants, such as *N. benthamiana*, whose entire genome sequence information is not well known. Carotenoids are essential for protecting the photosynthetic system against photooxidative damage (Bartley and Scolnik, 1995; Niyogi, 1999). Null mutation of the *LytB* gene homolog *IspH* in *Arabidopsis* resulted in an albino phenotype and severely impaired chloroplast development (Ahn and Pai, 2008; Jeon et al., 2010). Since ROS production is highly correlated with HR, the inhibition of HR PCD in *NbLytB*-silenced plants was suggested to be due to a decrease in chloroplast ROS production. However, the abnormalities in chloroplast morphology and the decrease in photosynthetic efficiency observed in *NbLytB*-silenced plants were very similar to those observed in *NbPDS*-silenced plants in which suppression of HR PCD was not observed (**Supplementary Figure 3**). In addition, MEP inhibitor treatment suppressed HR PCD but did not reduce H_2_O_2_ production relative to that in the buffer-treated control plants (**Figures 4** and **5**). Taken together, these results suggest that HR PCD inhibition in *NbLytB*-silenced plants is more strongly associated with lower MEP pathway metabolite levels than with the amount of chloroplast-generated ROS.

IPP, used by prenyltransferases as a building block for the sequential elongation of hydrocarbon skeletons, is synthesized either *via* the cytosolic MVA pathway or the plastidic MEP pathway (Peñuelas and Munné-Bosch, 2005; Pulido et al., 2012). The importance of the cytoplasmic MVA pathway for the immune system of animals has long been emphasized because isoprenoids produced from the MVA pathway are converted into various secondary lipids and used for the post-translational modifications of proteins (Buhaescu and Izzedine, 2007; Akula et al., 2016). However, in plant immune responses, it is not yet known whether each of the two independent IPP production pathways have distinct immune response mechanisms. According to the results of the current study, *Bax* overexpression- and R–Avr interaction-induced HR-related PCD was dependent on plastid MEP pathway metabolites, and did not appear to be complemented by cytosolic MVA pathway metabolites. Unlike mammals, most known plant pattern recognition receptors (PRRs) are localized to the plasma membrane (PM). Because protein post-translational modifications are important for PM localization, it will be interesting to observe the role of MEP-derived IPP in the subcellular localization of these key PRRs (Macho and Zipfel, 2014). Among plant BAGs, BAG6 is localized to vacuoles and induces cell death, unlike other BAGs (Kang et al., 2006). Future studies directed at determining whether the reduction of plastid-derived IPP affects intracellular organelle targeting of specific defense-related proteins and PCD regulatory proteins would help elucidate the role of plastid-derived IPPs in plant immunity.

Overexpression of BI-1 in *Arabidopsis thaliana* suppresses Bax-induced cell death without inhibiting ROS production (Kawai-Yamada et al., 2004). Further studies showed that BI-1 partially inhibits Bax-induced cell death by regulating Ca^2+^ homeostasis of the ER at a stage following ROS generation (Ihara-Ohori et al., 2007). These results are very similar to those obtained using FOS-treated plants, in which Bax-induced HR is suppressed even in the presence of ROS generation (**Figure 4**). The ER stress response is closely related to *R* gene-mediated HR (Williams et al., 2014). Silencing of ER chaperones involved in ER-quality control, such as calreticulins in *N. benthamiana*, compromises receptor-like protein Cf-mediated immune responses (Liebrand et al., 2012), and binding immunoglobulin protein (BiP)-silenced tobacco plants show reduced HRT-mediated HR (Moon et al., 2016). Together, these results raise the possibility that MEP-derived IPPs are linked with the ER stress responses to modulate HR-PCD. Future studies will be needed to determine whether the reduction of MEP-derived IPP induces a stress response in the ER.

Polyisoprenoids are secondary isoprenoid metabolites, and although their biological functions have not yet been clearly elucidated, it is assumed that they serve as lipid carriers in the process of protein glycosylation in plants (Surmacz and Swiezewska, 2011). *Arabidopsis* mutants with reduced biosynthesis of the polyisoprenoid dolicol show reduced protein glycosylation, increased sensitivity to darkness, and an early senescence phenotype (Zhang et al., 2008). The 3-hydroxy-3-methylglutaryl CoA reductase gene, an important regulator of the MVA pathway, is strongly expressed in tobacco leaves during the TMV resistance response (Kang et al., 1998), and the expression of two key enzymes in the MEP pathway, 1-deoxyxylulose 5-phosphate synthase and DXR, is increased in rice following treatment with a chitin elicitor (Okada et al., 2007). These results suggest that polyisoprenoids involved in plant defense are likely ‘mosaic compounds’ originating from both the MEP and/or MVA pathways (Skorupinska-Tudek et al., 2008). Further studies are needed to determine whether the reduction of MEP pathway-generating IPP caused by *NbLytb* silencing affects the production of specific polyisoprenoids and whether this results in an ER stress response associated with the accumulation of immature proteins in the ER.

In conclusion, these results demonstrate that the plastid MEP pathway is involved in plant defense responses, supporting the importance of the chloroplast-produced IPP in HR-PCD in plants. Further experiments are required to understand how this pathway is integrated with upstream and downstream pathways, and to obtain further insights into the complex regulatory mechanisms underlying PCD and disease resistance in plants.

## Data availability statement

All of the data supporting the results of this study can be found in either the article/**Supplementary Materials** together with the accession numbers of the nucleotide sequences deposited in GenBank (NCBI).

## Author contributions

SL and JP conceived and designed the study; SL performed VIGS and chemical inhibitor treatment experiments; SJ, CH, and JL performed cloning, RT-PCR, and TB staining. SL, SJ, CH, JL, and JP wrote the manuscript; SL, SJ, BC, and JP revised the manuscript. All authors contributed to the article and approved the submitted version.

## Funding

This work was supported by the KRIBB Initiative Program (KGM9942213 and KGM5372221) and the Basic Research Program of National Research Foundation of Korea grant (NRF-2017R1A2B4012820 and NRF-2020R1I1A2075335 to JP) of the Korean government (MSIP: Ministry of Science, ICT, and Future Planning).

## Acknowledgments

We thank Dr. Dinesh Kumar for providing the *pTRV1* and *pTRV2* constructs, Sir David Baulcombe for providing *Rx*, *PVX CP*, and *PVX CP* mutant clones, and Dr. Youn-il Park, who helped measure the photosynthetic efficiency of VIGSed plants.

## Conflict of interest

The authors declare that the research was conducted in the absence of any commercial or financial relationships that could be construed as a potential conflict of interest.

## Publisher’s note

All claims expressed in this article are solely those of the authors and do not necessarily represent those of their affiliated organizations, or those of the publisher, the editors and the reviewers. Any product that may be evaluated in this article, or claim that may be made by its manufacturer, is not guaranteed or endorsed by the publisher.
