# Supplementary material for: Plastid methylerythritol phosphate pathway participates in the hypersensitive response-related cell death in Nicotiana benthamiana
Source: Front Plant Sci. 2022 Oct 26;13:1032682. doi: 10.3389/fpls.2022.1032682 (PMC9645581; doi:10.3389/fpls.2022.1032682)
Supplement: Supplementary file 1 [file DataSheet_1.docx]

Supplementary Material

# Supplementary Figures and Tables

## Supplementary Figures


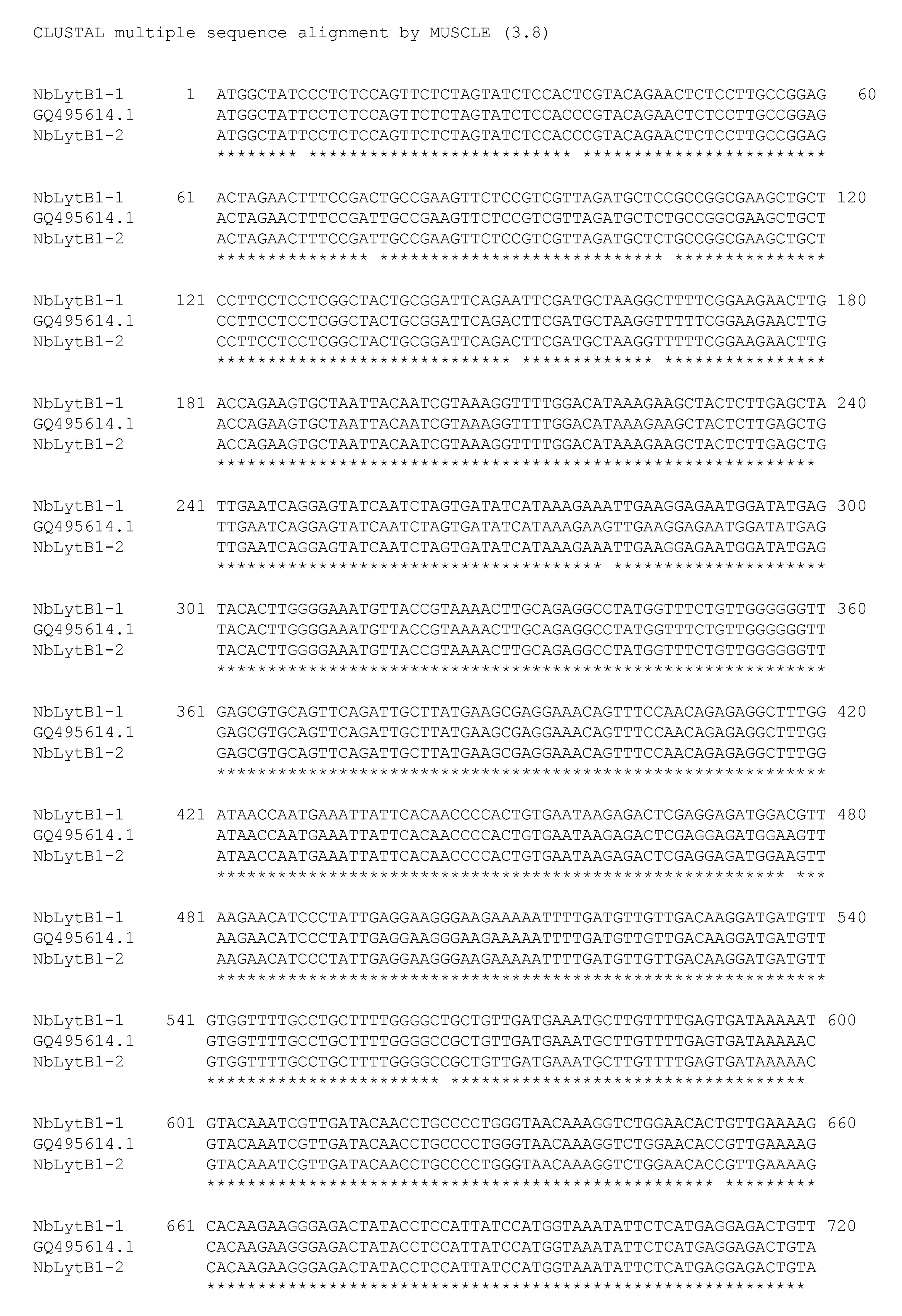

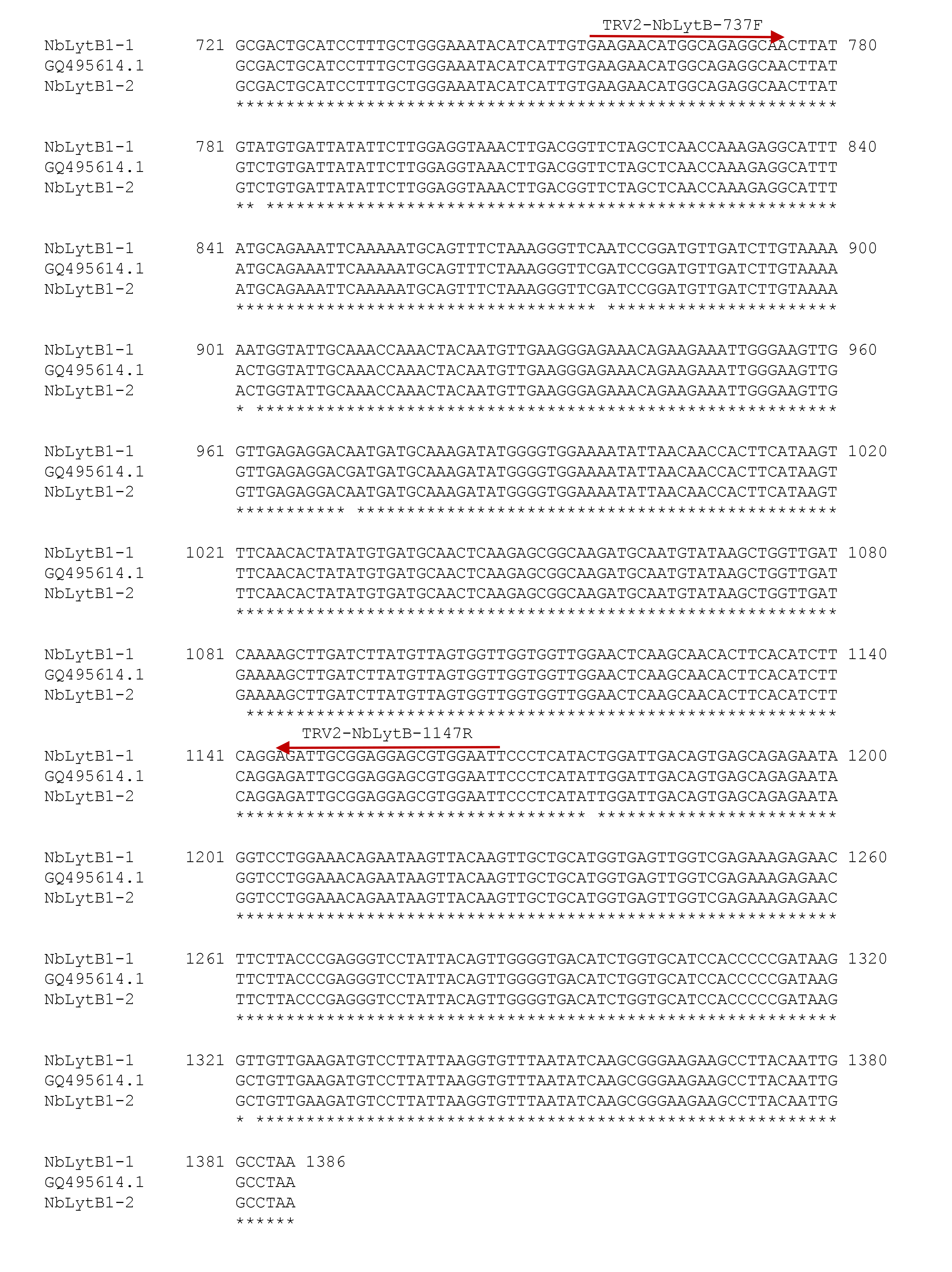


**Supplementary Figure 1.** Nucleotide sequence alignment of *Nicotiana benthamiana LytB* genes (*NbLytB1-1* and *NbLytB1-2*) and GQ495614.1 (downloaded from the NCBI nucleotide database). Asterisks indicate identical nucleotides. The sequence used to generate the virus-induced gene silencing (VIGS) construct is represented by red arrows, and the primers used to amplify this sequence are also indicated. The alignment was constructed using MUltiple Sequence Comparison by Long-Expectation (MUSCLE; <https://www.ebi.ac.uk/Tools/msa/muscle/>).


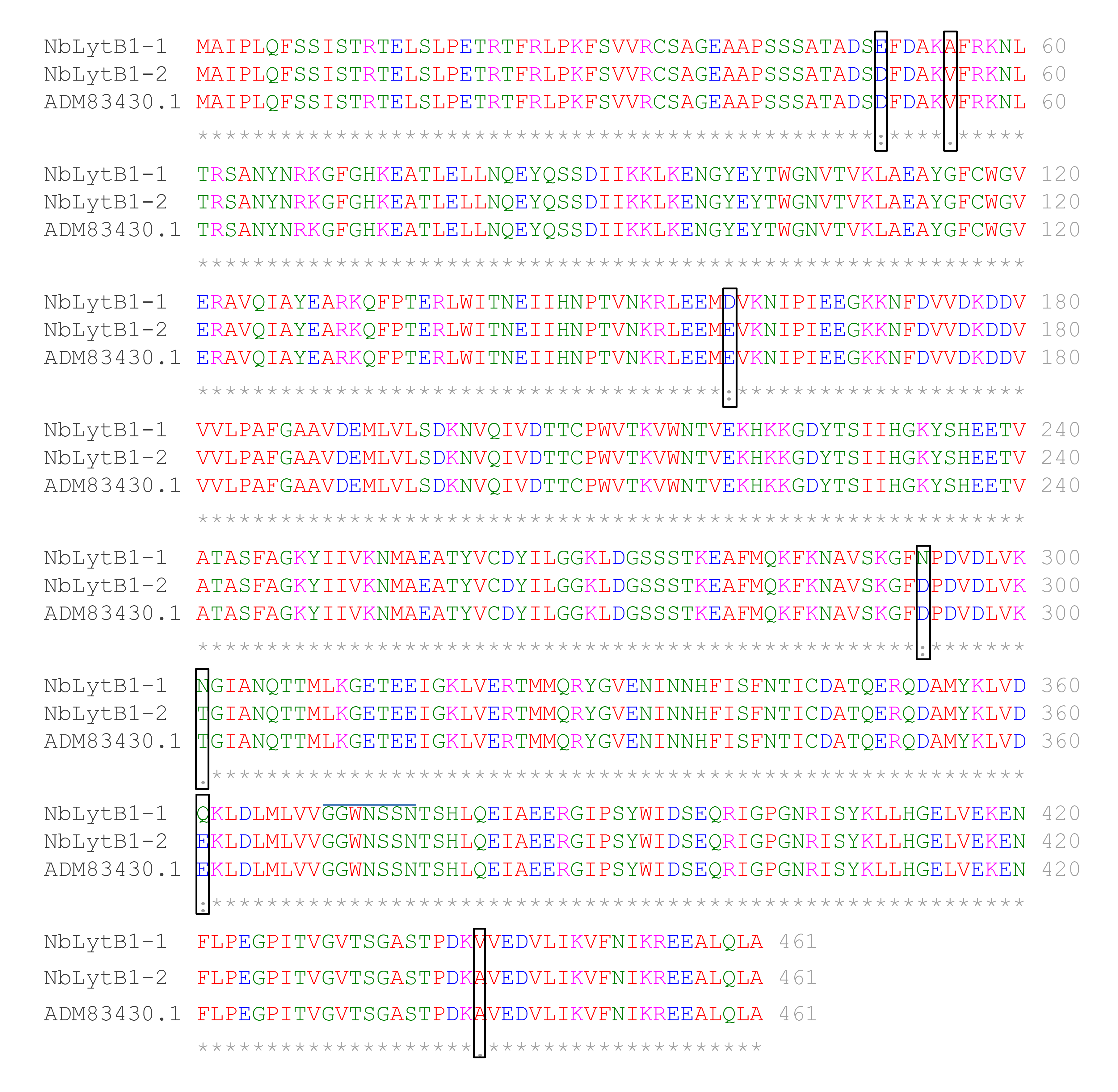


**Supplementary Figure 2.** Amino acid sequence alignment of NbLytB1-1, NbLytB1-2, and ADM83430.1 (downloaded from the NCBI protein database). Asterisks (*) indicate fully conserved residues; colons (:) indicate conservation between residues with highly similar properties (score >0.5 in the Gonnet PAM 250 matrix ([https://www.ebi.ac.uk/seqdb/confluence/display/JDSAT/ Bioinformatics+Tools+FAQ](https://www.ebi.ac.uk/seqdb/confluence/display/JDSAT/%20Bioinformatics+Tools+FAQ)); dots (.) indicate conservation between residues of weakly similar properties (score ≤0.5 or >0 in the Gonnet PAM 250 matrix). The alignment was constructed using MUSCLE (<https://www.ebi.ac.uk/Tools/msa/muscle/>).

**
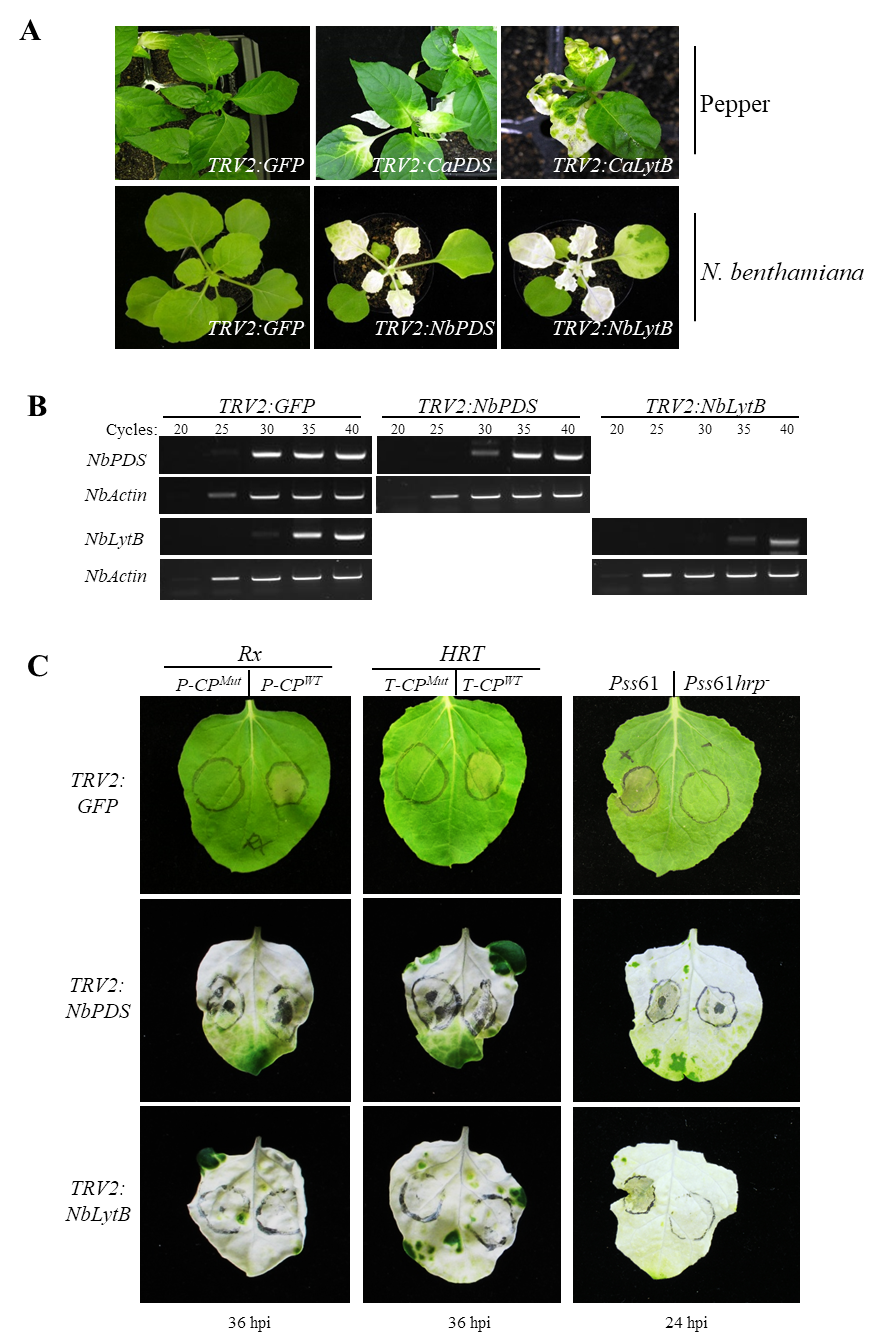
**

**Supplementary Figure 3.** *NbPDS*- and *NbLytB*-silenced plants exhibit similar morphological phenotypes but displayed different responses to HR-PCD induced by *Rx/CP*, *HRT/CP*, and *Pss*61. (A) *GFP*-silenced plants exhibited normal growth phenotype, whereas *NbPDS*- and *NbLytB*-silenced plants produced albino leaves. *TRV2:GFP* was used as a negative control, and *TRV2:NbPDS* was used as a positive control. Images represent the typical phenotypes of *GFP*-, *NbPDS*-, and *NbLytB*-silenced plants at 2 weeks after VIGS. (B) Relative expression levels of *NbPDS* and *NbLytB* estimated by semiquantitative RT-PCR in gene-silenced plants. *Actin* was used as an internal control. (C) Comparison of HR-PCD induced by *Rx/P-CP*, *HRT/T-CP*, and *Pss*61 in *GFP*-, *NbPDS*-, and *NbLytB*-silenced plants. The HR-PCD responses at infiltration sites were evaluated at the indicated time points, and pictures were taken. Three independent experiments were performed with similar results.

**
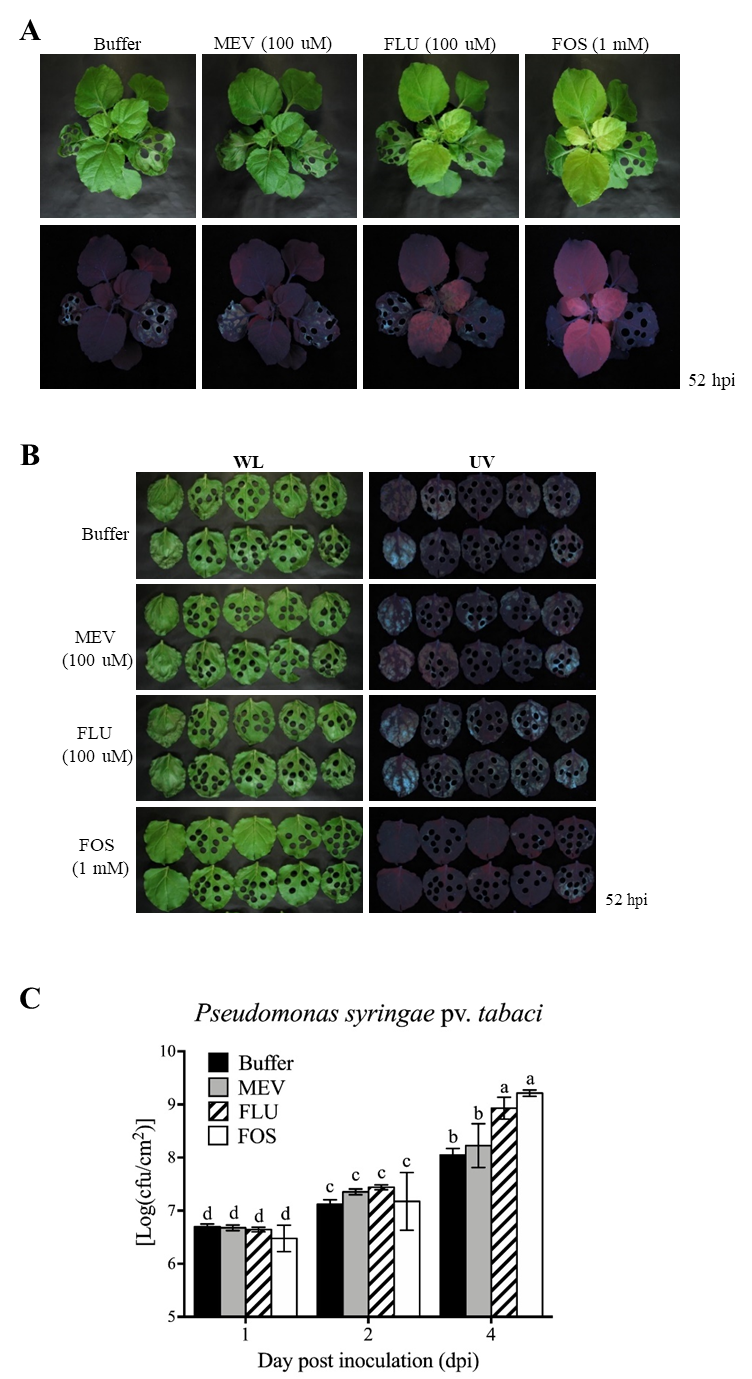
**

**Supplementary Figure 4.** Analysis of the effects of isoprenoid biosynthesis inhibitors on Bax-mediated HR in *N. benthamiana* plants. (A) Phenotypes of *N. benthamiana* leaves treated with various chemical inhibitors. Fully expanded leaves of 4-week-old *N. benthamiana* plants were treated with 0.01% ethanol (Buffer), 100 μM MEV, 100 μM FLU, or 1 mM FOS. Plants were photographed 5 days after the inhibitor treatment. (B) FOS inhibited Bax-mediated HR in *N. benthamiana*. Sixteen hours after inhibitor treatment, *Agrobacterium* expressing *Bax* was infiltrated into the inhibitor-treated leaves. Pictures were taken under white light (WL; left panel) and ultraviolet light (UV; right panel) at 52 hpi. Three independent experiments were performed with similar results. (C) Growth of *Pst* in inhibitor-treated plants. The leaves of each inhibitor-treated plant were inoculated with *Pst* and the bacterial inoculum was measured at 1, 2, and 4 dpi. Data represent mean ± SD of at least three biological replicates, with each replicate containing six leaf discs. Different letters indicate differences (*P* < 0.05; Student’s *t*-test). Three independent experiments were performed with similar results.

## Supplementary Table

| **Name** | **Forward primer (5’-3’)** | **Reverse primer (5’-3’)** |
| --- | --- | --- |
| L^1^-*NbLytB* | TTGGTGGTTGGAACTCAAGC | CGGATCGAACCCTTTAGAA |
| 5´-RACE |  | CCGGATCGAACCCTTTAGAA |
| SQ^2^-*NbPDS* | CACGCCCAACTAAACCATTG | AAGATTGCCCTCCAAGCATT |
| SQ-*NbLytB* | GCTTGAGTTCCAACCACCAA | CTGTTGCGACTGCATCCTTT |
| SQ-*NbActin* | TGGACTCTGGTGATGGTGTC | CCTCCAATCCAAACACTGTA |
| TMV MP | TGCTATAACCACCCAGGACG | CGACAGTAGCCTCCGAATCA |

**Supplementary Table 1.** List of primers used for the cloning and expression analysis of *NbLytB* and other genes.  ^1^,L=Library PCR; ^2^, SQ=semi-quantitative.
